# Simultaneous Use of Iron/Anticoccidial Treatment and Vaccination against Oedema Disease: Impact on the Development of Serum-Neutralising Antibodies, Hematinic and Anticoccidial Activities in Piglets

**DOI:** 10.3390/vaccines12091004

**Published:** 2024-09-01

**Authors:** Daniel Sperling, María Rodríguez, Nicolás Guerra, Hamadi Karembe, Anne-Kathrin Diesing, Alberto Manso, Laura de Frutos, Joaquín Morales

**Affiliations:** 1 Ceva Santé Animale, 10 Avenue de la Ballastière, 33500 Libourne, France; nicolas.guerra@ceva.com (N.G.); hamadi.karembe@ceva.com (H.K.); anne-kathrin.diesing@ceva.com (A.-K.D.); 2 Animal Data Analytics S.L., C/Dámaso Alonso 14, 40006 Segovia, Spain; maria.rodriguez@ada-animaldata.com (M.R.); alberto.manso@ada-animaldata.com (A.M.); laura.defrutos@ada-animaldata.com (L.d.F.); joaquin.morales@ada-animaldata.com (J.M.)

**Keywords:** Oedema disease, cystoisosporosis, toltrazuril, vaccination, IDA, haemoglobin, gleptoferron

## Abstract

Oedema disease (OD) in weaned piglets is caused by shigatoxigenic *Escherichia coli* (STEC), which produces the Stx2e toxin. The disease is controlled by early vaccination (for example, with Ecoporc Shiga^®^). Iron-deficiency anaemia (IDA) and cystoisosporosis are the most common clinical conditions in piglets. These conditions are managed mainly by the intramuscular injection of iron and application of toltrazuril (for example, Forceris^®^). In the present study, we sought to evaluate any effect on the efficacy of OD vaccination and iron/anticoccidial treatment resulting from a simultaneous application. An evaluation was carried out by measuring the development of neutralising antibodies against the Stx2e toxin, hematinic indices and oocysts shedding. Six litters from Stx2e-antibody-negative sows were included in the study, with 12 piglets in each litter. The piglets were randomly allocated into two groups on their second day of life (DOL): (T1) iron/anticoccidial treatment and vaccine were administered on different days, and (T2) products were administered simultaneously. Blood samples were collected to determine the levels of serum-neutralising antibodies, haemoglobin and haematocrit. Faecal matter was examined for the presence of oocysts of *Cystoisospora suis*. No differences were found between the two groups in terms of the development of neutralising antibodies. The levels of haemoglobin and haematocrit were lower (*p* < 0.05 and *p* = 0.08, respectively) when iron/anticoccidial treatment and vaccine were applied simultaneously but within the optimal range, based on current interpretive criteria for IDA. Oocysts were not detected in the faecal samples from the animals in either group. In conclusion, we found that, under the conditions of our study, the efficacy of OD vaccination and iron/anticoccidial treatment was not affected by the simultaneous use.

## 1. Introduction

Oedema disease (OD) is a form of enterotoxaemia caused by strains of Shiga-toxin-producing *Escherichia coli* (*E. coli*) (STEC). The disease occurs in pigs worldwide and is characterised by acute systemic enterotoxaemia. OD is known to result in high levels of mortality on affected farms; however, a subclinical form of the disease has also been described [1,2]. In a large-scale study carried out on industrial farms in Germany, the prevalence of *E. coli* encoding for Stx2e (STEC-2e) was found to be as high as 35.1% (95% confidence interval (CI) 31.0–39.1%) in pens, and 53.5% (95% CI 44.4–63.6%) on farms [3]. OD mainly affects piglets during the nursery period (the post-weaning stage of production). Vaccination is now considered one of the most important tools for protecting piglets against OD because it avoids the use of antimicrobials that may trigger selection for antimicrobial resistance. Vaccination also avoids the use of zinc oxide, which is considered to present an environmental risk [4,5] and has recently been banned in EU member states [6,7]. Modified recombinant Stx2e-based toxoid vaccines have proven effective not only against clinical manifestations of OD, including mortality, but also subclinical infection, which is characterised by impaired zootechnical performance and immunosuppression [7,8]. Vaccination of suckling piglets via intramuscular routes has been shown to induce the production of protective Stx2e-neutralising antibodies [9]. Vaccine efficacy has also been demonstrated in laboratory and field studies by the detection of neutralising antibodies after immunisation, and by significant reductions in morbidity and mortality in vaccinated pigs compared with control animals [2,9,10]. To protect piglets in the sensitive period of life after weaning at 21–28 days of life (DOLs) and onwards, it is now a well-established practice to vaccinate around DOL 4.

Iron-deficiency anaemia (IDA) is a global public health problem, not only for humans but also for animals. IDA is the most common mineral deficiency in neonatal piglets; such animals are now routinely subjected to parenteral iron supplementation during the first days of life [11]. In pigs, IDA has been associated not only with metabolic stresses such as an impaired immune response, increased sensitivity to infections, and impaired zootechnical performance but also behavioural disorders [12,13,14]. *Cystoisospora suis* is the causative agent of cystoisosporosis, a disease which causes diarrhoea. In piglets, cystoisosporosis is the most frequently occurring form of parasitic infection, with high prevalence rates reported in swine-producing countries [15].

It is now common practice worldwide for piglets to receive iron parenterally during their first days of life to prevent IDA and top up their limited stores of iron [11]. Interventions for the control of cystoisosporosis also take place during the first days of life [11,16], and vaccination against OD is also typically administered during the neonatal period. In the swine industry, there is an interest in vaccinating piglets at the same time as other procedures such as castration, tail docking, and teeth clipping are carried out. This reduces labour inputs and costs and minimises discomfort and handling stress, which promotes the welfare of animals [17,18].

Several studies have confirmed that vaccines underperform when certain conditions apply at the time of vaccination, including undernutrition and impaired health status resulting from IDA; with respect to the latter, the importance of iron status in vaccine response has already been reported [19]. In humans, the application of iron at the time of vaccination has been shown to improve immune response after administration of important vaccines, including the combined diphtheria and tetanus vaccine, the pneumococcal vaccine, and the measles vaccine [20,21,22].

The development of neutralising antibodies is frequently used to characterise vaccine take and protection (a correlate of protection (CoP)) [23].

The aim of the current study was to evaluate the effect of simultaneous application of iron/anticoccidial treatment and OD vaccine on the efficacy of vaccination in young piglets based on an evaluation of neutralising antibodies. We also evaluated parameters indicating the efficacy of the iron/toltrazuril product, including hematinic parameters, parasite-infection status, and the zootechnical performance of piglets.

## 2. Materials and Methods

### 2.1. General Husbandry and Management of Animals

The study was conducted between May 2022 and June 2022 on a commercial breeding-herd farm housing 595 sows in Spain. Sows and piglets were housed, fed, and managed according to the recommendations of Directive 2010/63/EU concerning standards of animal husbandry, hygiene, nutrition, welfare, housing, and care. The lactation barn included 12 identical rooms, each containing 12 cages. Six weeks prior to expected farrowing, 15 randomly selected sows (DNA genetics; third to sixth parturition) were sampled to check for the presence of serum-neutralising antibodies against Stx2e toxin. Six sows which tested negative were selected for the study. Seven days before farrowing, sows were assigned to a gestation barn on the same commercial farm, which was in line with standard farm practice. They were then moved to experimental lactation rooms, where they were assigned individual cages based on parity. Piglets (DNA sow × Pietrain boar) were weaned at DOL 28, and the study ended after DOL 60.

Within 48 h of birth, the piglets were individually weighed and identified with ear tags. Litters were equalised at 12 piglets/litter by cross-fostering among sows that were farrowed on the same day. The piglets included in the study had a minimum weight of 1 kg at inclusion and were clinically healthy, with similar sex ratios for each investigated group. Piglets were provided with high-quality, digestible creep feed from day 10 of lactation onwards and were weaned at DOL 28. Piglets were kept in the same litters, and treatment was randomly allocated within each litter by computer to give a randomised block design. Within each litter, there were six piglets per treatment.

At weaning (DOL 28), piglets were housed in climate-controlled nursery rooms with 12 pens per room, each with a partially slatted floor. The pens included one feeder and one waterer. Piglets which showed signs of illness, injury, or poor condition were excluded. Non-medicated pre-starter feed (commercial diet) was offered from weaning (DOL 28) to 14 days post-weaning. Non-medicated starter feed (commercial diet) was offered from 15 to 42 days post-weaning. All feeds were provided to the piglets ad libitum in pellet form from feeders placed inside the pens. Non-medicated water was also provided ad libitum from the drinkers. The environmental conditions during the study (temperature and ventilation rate) were automatically controlled according to the age of the piglets.

### 2.2. Products

The intramuscular formulation contained 30 mg/mL toltrazuril and 133 mg/mL iron (as gleptoferron) in a combo product (Forceris^®^, Batch number: 536BB, Ceva Santé Animale, Libourne, France) with a dosage of 1.5 mL per piglet. Vaccination was carried out using inactivated recombinant Stx2e toxoid vaccine (Ecoporc Shiga^®^, Batch number: 1481121C, Ceva Santé Animale, Libourne, France), 1 mL. Both products were administered according to their respective Summary of Product Characteristics (SPC).

### 2.3. Experimental Design, Blinding, and Treatments

**Figure 1:**
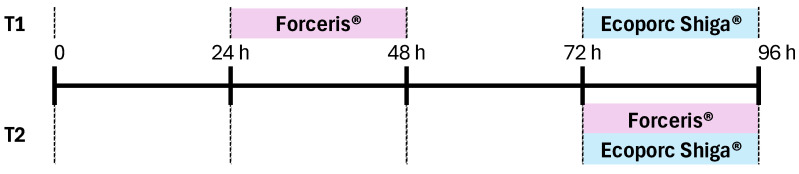
Two experimental groups were established on the second day of life (DOL). Piglets in group T1 were administered Forceris^®^ and Ecoporc Shiga^®^ on different days (24–48 and 72–96 h after birth, respectively); piglets in group T2 were administered Forceris^®^ and Ecoporc Shiga^®^ simultaneously (72–96 h after birth).

We followed good clinical practice by using a parallel, randomised, observer-blinded, experimental block design to compare the two different treatment protocols. The experimental products were administered by a dispenser with a treatment allocation list. This dispenser did not participate in the rest of the study. A total of six litters from Stx2e-antibody-negative sows were included, and 12 piglets from each litter (72 piglets in total) were used in the study. The animals were randomly assigned to one of the two groups on their second day of life. Piglets in the T1 group were administered Forceris^®^ and Ecoporc Shiga^®^ on different days (24–48 h and 72–96 h after birth, respectively); those in the T2 group were administered Forceris^®^ and Ecoporc Shiga^®^ simultaneously (72–96 h after birth) (Figure 1). Early application of iron-based products is mandatory and standard practice nowadays as most piglets are born already anaemic and fatal IDA will develop without iron supplementation within the first week of age [24]. Moreover, OD causes significant losses due to its high mortality rate. The case mortality rate ranges from 50% to over 90% on affected farms, with no effective treatment available once clinical symptoms develop [1]. For ethical reasons and because there was a lack of effective rescue measures for both disease conditions, the control group (non-medicated and non-vaccinated) was not included in the study [25].

### 2.4. Serum Neutralisation Test

The development of serum-neutralisating antibodies in piglets and the proportion of animals that exceeded the established protective titre (>15) were evaluated using a non-commercial, validated in-house serum neutralisation test (SNT) by laboratories of Ceva Innovation Center GmbH, Dessau-Roßlau, Germany, developed based on the adopted protocol described by Pirro and colleagues [26]. A 4 mL blood sample was collected from each of the piglets (36 piglets/treatment group; 72 piglets in total) at DOL 28 and DOL 60. The tubes were centrifuged, and the serum samples (1 mL) were collected, transferred to Eppendorf tubes (Merck, Darmstadt, Germany), and frozen at −20 °C. Frozen samples were then transferred to the laboratory for testing. Dilution series with cell culture medium (DMEM (Cytiva, Logan, UT, USA), 10% FBS (Capricorn, Düsseldorf, Germany)- previously heat-inactivated, 2 mM GlutaMax (Thermo Fischer, Cambridge, UK) and 1% Penicillin/Streptomycin (Cytiva, Logan, UT, USA)) in a ratio of 1:2 were prepared from the piglet sera in the 96 well plates. A positive control (reference pig serum adjusted to a protective titer of 15) and a negative control (reference serum from confirmed Stx2e seronegative pig) were included in the test and treated in the same way. The dilutions were incubated at an atmosphere of 5% CO_2_ at 37 °C in a cell culture incubator with Stx2e at a constant volume and concentration. If neutralising antibodies were present in the investigated sera, they would effectively bind the toxin. A suspension of Stx2e sensitive Vero cells (ATCC CRL 1587) was added to preincubated 96 well plates. The serum samples were incubated for 4 days on Vero cells. Subsequently, the cell cultures were incubated with a chemical which is metabolised by living cells into a yellow dye (the cell proliferation reagent WST-1, Roche, Mannheim, Germany). Using this method, the viability of the cells could be measured photometrically. The relative viability of cells with serum was calculated with respect to the negative control (set to 100% viability) and the toxin control (set to 0% viability). Additionally, based on the negative control and toxin control, a 50% viability value was calculated. The reciprocal value of the last serum dilution, which had a relative viability of ~50%, was defined as the titer, i.e., the final result. The determination was carried out using two replicates per sample. The titration curve of the positive control was used as a reference.

### 2.5. Hematinic Parameters and Parasitology Examination

Hematinic parameters (haemoglobin and haematocrit) were evaluated. To that purpose, at least 0.4 mL of blood per piglet was collected from jugular veins on days 28 and 60 of life and transferred to EDTA tubes. Blood samples were immediately labelled, stored at +5 °C and shipped for analysis within 24 h of collection. The levels of haematocrit and haemoglobin were then determined by Labopat (Segovia, Spain). 

Piglets were classified into three categories according to their haemoglobin (Hb) concentrations: optimal iron status (Hb > 11 g/dL), subclinical iron deficiency (Hb 9–11 g/dL, buffer zone), or anaemia (Hb < 9 g/dL) [27]. The percentage of anaemic piglets was then calculated.

In addition, parasitological examination of individual faecal samples was performed using a previously described flotation method [28]. This involved a routine and accredited technique which was applied in the laboratory involved in the study. Samples were collected from individual piglets directly from the rectum on day 21 of life. In total, 72 faecal samples were evaluated, 36 from each treatment group. Faecal analyses were also conducted by Labopat (Segovia, Spain).

### 2.6. Performance Evaluation

The piglets were weighed individually on days 0, 21, and 60 of the study. The average daily gain (ADG) of the piglets was calculated for the following periods: 0–21 days, 21–60 days, and 0–60 days.

Any piglet mortality pre- or post-weaning, as well as the cause of death/cull, was investigated with a veterinary necropsy. All such necropsies were limited to establishing a probable cause of death.

### 2.7. Statistics

All researchers involved in this study (i.e., those performing daily procedures on the farm, those involved with data collection, and those involved with data analysis) remained blind until the final data analyses had been completed. The statistical software used was SAS v9.0 (Cary, NC 27513-2414, USA). The individual piglet was the experimental unit. The results of SNT were analysed considering both detectable neutralising antibodies (>0) and proportions of animals with protective titre (≥15). These variables were analysed as binary variables (each piglet with antibodies >0 and titre ≥15 was assigned a value of 1; otherwise, it was assigned a value of 0) using the chi-square test (proc FREQ of SAS). The percentage of piglets categorised as sub-anaemic or having an optimal concentration of haemoglobin was also analysed as a binary variable using the chi-square test (proc FREQ of SAS). For parameters measured multiple times (repeated measures), a linear mixed-effects model was used (proc MIXED of SAS). The fixed effects were the treatment group, the time of sampling, and their interaction. Body weight (birth weight) was included as a covariate, and sow (blocking variable) was the random effect.

For the evaluation of parasitological results (presence of oocysts), a linear model was used, including the fixed effects of the treatment group and litter (blocking variable). Initial body weight was also included as a covariate. The model used was ANOVA.

In all the statistical analyses, *p* ≤ 0.05 was considered statistically significant, while 0.05 < *p* ≤ 0.10 was considered a trend.

## 3. Results

### 3.1. Serum Neutralisation

The serum neutralisation test (SNT) showed no differences between treatments in the case of either parameter, i.e., seroconversion after vaccination or proportion of piglets which reached protective titre (Table 1); this was true for determinations made at DOL 28 and at DOL 60 (*p* > 0.05). It should be noted that time had influence on the number of animals with detectable neutralising antibodies (11.5% increase, *p* = 0.0157), and the proportion of animals with protective titre ≥ 15 (24.5% increase, *p* = 0.0004).

### 3.2. Haematinic Indices

Table 2 shows the proportions of animals with different statuses according to the current categorisation of IDA in piglets based on Hb levels. No piglets with a Hb concentration below 9 g/dL (anaemic) were detected during the study. Animals with Hb concentrations between 9 and 11 g/dL were considered sub-anaemic (buffer zone), and animals with concentrations above 11 g/dL were considered optimal.

There were no differences between treatments in any case; this was true for determinations made at DOL 28 and DOL 60 (*p* > 0.05). Notably, time did have an effect, with the number of piglets in the buffer zone increasing by approximately 30% between day 28 and day 60, while the percentage of piglets with the optimal concentration decreased by the same percentage (*p* < 0.001).

Further examination of individual Hb levels was then carried out, and exploratory data analysis detected one outlier belonging to the T1 group (3.2 g/dL). Similarly, exploratory data analysis of haematocrit detected two outliers. The first one was found in the haemoglobin-outlier animal mentioned above and, thus, belonged to T1 (9.5%). The second outlier detected belonged to T2 (6.46%). These animals were removed for further analysis.

Table 3 shows a comparison between administration protocols with respect to Hb and haematocrit concentrations in individual animals at different times. The concentrations of haemoglobin were lower (*p* < 0.0274) when treatments were administered simultaneously but were still within the optimal category (11.99 g/dL), expressed as the mean. The concentrations of haematocrit also tended to be lower (*p* = 0.079) in the simultaneous-treatment group. Concentrations of both Hb and haematocrit decreased between day 28 and day 60 (*p* < 0.05) in both treatment groups. The interactions of both parameters with respect to the administration protocol and sampling day were not significant (*p* > 0.05).

### 3.3. Evaluation of Presence of C. suis Oocysts 

Oocysts of *C. suis* were not detected in any sample during the course of the present study.

### 3.4. Growth Performance

The choice of administration protocol did not affect (*p* > 0.05) growth performance (ADG) during the lactation period (from DOL 0 to DOL 21); consequently, body weights (BWs) at DOL 21 were similar in both experimental groups (Table 4).

However, although the ADG also remained similar in both groups between day 21 and day 60 (*p* > 0.05), the BW on day 60 tended to be higher (*p* = 0.1) in piglets which received both treatments simultaneously. Total ADG (from DOL 0 to DOL 60) also tended to be higher (*p* = 0.1) in the animals in the simultaneous-treatment group.

### 3.5. Mortality

Three piglets died during the study period: two in the lactation period (both on the second day of life) and one in the nursery period (on the thirty-first day of life). All three belonged to the T1 group, which received treatments on different days. None of them showed clinical signs of OD, and disease was excluded during the necropsy.

## 4. Discussion

OD is caused by *Escherichia coli*-producing Stx2e toxin (STEC), which is one of the major pathologies in nursery pigs, and it may cause considerable losses on affected farms [4,29]. The morbidity and mortality caused by clinical OD have led to the increased use of antibiotic medications and critically important antimicrobials (CIAs) such as colistin and fluoroquinolones, with consequent pressure from resistance development [5,7,9]. Even more concerning is the fact that antibiotics are generally not recommended in such cases. Because toxins in the Stx family are encoded by phages, antibiotic therapies may induce an SOS (cellular stress) response, promoting the release of toxins and increasing the severity of clinical disease [30,31]. As a result, strategies for the control of OD using vaccination with toxoids based on recombinant Stx2e have increased in importance.

In piglets, OD occurs mainly in the nursery stage of development, during the post-weaning period of production. Early vaccination of piglets at around the fourth day of life is, therefore, crucial to provide protection in the sensitive period after weaning. In the swine industry, the first week of life is also characterised by different treatments such as iron administration and metaphylactic anticoccidial product application, as well as management procedures such as castration, tail docking, and teeth clipping [17,18,32].

Currently, there are limited data on immune responses when vaccination is administered simultaneously with other pharmaceutical products. In the present study involving piglets, we sought to investigate neutralising antibodies as immunological responses to early vaccination against OD when a product containing iron and toltrazuril was administered during the same treatment session. The effect of iron/toltrazuril-based treatment was investigated at the same time. We then compared these responses with those observed in piglets who received vaccines and pharmaceutical products on different days. Under the conditions of the present study, we found that the simultaneous application of an iron/toltrazuril-based product and vaccination against OD provided a similar rate of animals with the presence of antibodies against Stx2e toxin and animals with antibody levels equal to or exceeding established protective titer (≥15). We found that the inactivated vaccine elicited strong humoral immune responses, as measured by SNT, with subsequent increases in the number of responding animals and animals exceeding protective titre, regardless of the choice of treatment protocol. Overall, only four animals did not exhibit any antibody development: three in the T1 group and one in the T2 group. This may represent normal variation in population.

The serum neutralisation test was used to assess the humoral immune response to the Stx2e toxin. SNT is regarded as the gold-standard functional serologic test. Neutralising antibodies are considered an effector part of the humoral response of the adaptive immune system; they protect sensitive cells by neutralising the biological effects of the Stx2e toxin after its release into blood circulation [24,33,34]. In the case of OD, the development and presence of neutralising antibodies are considered a correlate of protection (CoP); this was used in the development of the vaccine and continues to be employed for the evaluation of vaccination compliance in the field [3,35,36]. Different modifications of the toxin neutralisation (TN) test have been used for antibody limit testing in the development of other vaccines, e.g., in human medicine and diphtheria potency testing of toxoid vaccine [24,37]. In addition, animals with detectable Stx2e antibodies have been found to be protected based on the results of challenge trials during the development of vaccines (Ceva internal data, [38]). In the present study, two time points (DOL 28 and DOL 60) were used for evaluation, representing the onset of immunity (OI) and the end of the nursery period, respectively, when the animals are most sensitive to clinical OD.

In the field, antibiotics and other pharmaceutical products such as toltrazuril and iron are sometimes administered to pigs simultaneously with vaccines. It is, therefore, reasonable to assess the effects of such simultaneous administration on vaccination efficacy. A number of such assessments involving various antibiotics have been carried out by researchers. The authors of [39] found that some classes of antibiotics (ATB) with immunomodulating properties may influence the immune response to simultaneously applied vaccines. Antimicrobial therapy was found to modulate both cell-mediated and humoral post-vaccinal immune responses in pigs, and both positive and negative influences were described [39]. To the best of our knowledge, no such information concerning toltrazuril in piglets has previously been obtained. In the present study, we found that TZL did not influence vaccination response when this was measured based on the development of serum neutralisation antibodies. A previous study involving poultry suggested that therapeutic medication with toltrazuril does not interfere with the development of immunity and that medication with toltrazuril should be useful for treatment after vaccination with live anticoccidial vaccines, leading to an enhanced immune response [40]. The proposed mechanism was that, in treated chickens, the intracellular stages of the parasite, which were damaged by TZL stimulated the immune system of the host for a prolonged period [40]. In the present study, we did not observe any enhanced response when TZL was administered simultaneously with vaccine because the type of vaccine and the disease were both different; moreover, infection by *Cystoisospora suis* was not confirmed by parasitological examination during the present study.

Contrarily, the influence of iron on both immunity and vaccination efficacy has been well reported, especially in studies of human medicine. IDA at the time of vaccination has been associated with decreased response to different types of vaccines, and primary response to some vaccines has been improved by simultaneous supplementation with iron [41]. Piglets are born with limited iron stores, and many are born in an anaemic state (Hb levels < 9 g/dL) [24,42]. The foetal reserves of iron in newborn piglets are generally low, and this contributes to overall iron deficiency when Fe administration is delayed [42]. Early parenteral administration of iron is crucial for future development and growth in piglets. Such administration may also promote a proper response to early vaccination. On the other side, no information on the possible effect of vaccination and consequent immune stimulation on the pharmacokinetics/pharmacodynamics of iron and toltrazuril-based products is currently available. The metabolism of iron injected intramuscularly, where cells of the immune system (reticuloendothelial cells) play the role, is not properly understood. In the present study, however, we did not observe any differences in haematinic indices between the two treatment-protocol groups. No anaemic piglets were recorded, and similar proportions were recorded for both optimal and buffer-zone categories. Considering the mean Hb status of individual animals on the 28th day of life, we found that concentrations in the simultaneous-application group (T2) tended to be lower (*p* = 0.087) but still exceeded the threshold for the optimum category based on the current classification of IDA in piglets (>11 g/dL) [27]. In light of this, we may say that the clinical relevance of such a difference is probably negligible. The same pattern was observed for HMT on DOL 28. For Hb and HMT, decreases in concentrations over time were observed in the two treatment-protocol groups (*p* < 0.05). In the present study, we found that age significantly influenced Hb and HMT levels, in line with the findings of previously published studies showing that the composition of feed after weaning plays an important role after an initial drop following peak concentration resulting from parenteral Fe application. The values obtained at the end of the present study were also within a recently proposed reference range for nursery pigs [43]. However, with respect to the hematinic parameters which characterise iron storage and iron saturation capacity, considered early IDA indicators, we found no differences between our two treatment-protocol groups. In light of this, we may say that the pharmacodynamics of the iron part (hematinic indices) were not negatively influenced by simultaneous application.

Zootechnical parameters play a key role in animal performance, and these parameters include factors that may be negatively affected by pathogens but are also not effective in veterinary control programmes. As expected, the choice of administration protocol did not significantly influence zootechnical performance or growth in piglets during the observational period. For the lactation period from birth to 21 days, the ADG results were similar in the two study groups. Consequently, the BW values on day 21 were also similar. However, the BW on day 60 and the global ADG (from day 0 to day 60) tended to be higher in the animals which were administered treatments simultaneously. 

In conclusion, according to the results of our study, we found that simultaneous vaccination against OD with a toxoid vaccine and the administration of an iron/anticoccidial (TZL) product did not negatively impact the humoral response, as measured by the serum neutralisation test.

In addition, the application of iron/toltrazuril-based treatment was not influenced by vaccination at the same time, as measured by a panel of haematinic indices and parasitological evaluation. Our study provides pilot information of clinical interest because both treatments (iron administration and vaccination against OD) are indicated to be administered at the same moment of piglets’ lives to prevent clinical signs and possible mortality caused by OD and the development of IDA. This also applies to the metaphylactic treatment of cystoisosporosis on positive farms, where the early application of TZL is vital for effective treatment. Indeed, early application may be seen as crucial for effective protection against all conditions. As previously discussed, the early administration of iron to piglets can improve the efficacy of vaccination due to the resulting improvement in IDA status.

More practically, interest in combining different treatment protocols on farms is based on reducing the number of manipulations and handling of very young piglets who are already handled often during the neonatal period for purposes such as navel-cord care, teeth clipping, tail docking, ear notching for identification, castration of males, and litter transfer. Combined treatments can reduce stress in animals and improve their welfare, while, at the same time, reducing human labour requirements [44].

## 5. Conclusions

According to the results of our study, simultaneous vaccination against OD with a toxoid vaccine and the administration of an iron/anticoccidial (TZL) product did not negatively impact the humoral response of vaccinated animals, as measured by the serum neutralisation test. The application of iron/toltrazuril-based treatment was not influenced by vaccination at the same time, as measured by a panel of haematinic indices and parasitological evaluation (*Cystoisospora suis* oocyst shedding). The presented study provides pilot information of clinical interest because both treatments (iron/anticoccidial treatment administration and vaccination against OD) are indicated to be administered at the same moment of piglets’ lives to prevent clinical signs and possible mortality caused by OD and the development of IDA.

## Figures and Tables

**Table 1:** Proportions (%) of animals with detectable neutralising antibodies of both ≥ 0 and ≥ 15 on the 28th and 60th days of life when vaccine and iron/anticoccidial treatment were administered on different days (T1) or simultaneously (T2).

Treatment		Detectable Neutralising Antibodies (≥0) (%)	Detectable Neutralising Antibodies (≥15) (%)
Day 28	T1—Different days	87.88 ^b^	69.70 ^b^
	T2—Simultaneously	83.33 ^b^	63.69 ^b^
Day 60	T1—Different days	96.97 ^a^	90.91 ^a^
	T2—Simultaneously	97.22 ^a^	91.67 ^a^
P treatment		0.6549	0.7161
P time		0.0157	0.0004
P treatment × time		0.0983	0.0047

P treatment represents the *p*-value of the treatment as fixed effect. P time represents the *p*-value of the time of sampling as fixed effect. P treatment × time represents the *p*-value of the interaction between the treatment and the time as fixed effect. a, b: Different letters in the same column indicate significant differences in the interaction between treatments and time (*p* ≤ 0.05).

**Table 2:** Proportions of piglets categorised as sub-anaemic or having optimal haemoglobin concentration (9–11 g/d and above 11 g/dL, respectively) on days 28 and 60 of life when vaccine and iron/anticoccidial treatment were administered on different days (T1) or simultaneously (T2).

Treatment	Buffer Zone (%)	Optimal (%)
Day 28		
T1—Different days	0.00	100.00
T2—Simultaneously	5.56	94.44
Day 60		
T1—Different days	30.30	69.70
T2—Simultaneously	36.11	63.89
P treatment × time	<0.0001	

Buffer zone = subclinical iron deficiency (Hb 9–11 g/dL). Optimal = optimal iron status (Hb > 11 g/dL). P treatment × time represents the *p*-value of the interaction between the treatment and the time as a fixed effect.

**Table 3:** Concentrations of haemoglobin (g/dL) and haematocrit (%) detected in blood samples of piglets on days 28 and 60 of life when the vaccine and iron/anticoccidial treatment were administered on different days (T1) or simultaneously (T2).

Treatment	Haemoglobin (g/dL)	Haematocrit (%)
Day 28		
T1—Different days	13.18 ^a^	40.21 ^a^
T2—Simultaneously	11.44 ^b^	39.45 ^a^
Day 60		
T1—Different days	12.84 ^c^	36.30 ^b^
T2—Simultaneously	11.15 ^c^	35.32 ^b^
SEM	0.203	0.548
P treatment	0.0274	0.0792
P time	<0.0001	<0.0001
P treatment × time	0.8324	0.8199

P treatment represents the *p*-value of the treatment as a fixed effect. P time represents the *p*-value of the time of sampling as a fixed effect. P treatment × time represents the *p*-value of the interaction between the treatment and the time as a fixed effect. a, b: Different letters in the same column indicate significant differences in the interaction between treatments and time (*p* ≤ 0.05). SEM: standard error of the mean.

**Table 4:** Body weight (BW, kg) and average daily gain (ADG, g/day) of piglets when vaccine and iron/anticoccidial treatment were administered on different days (T1) or simultaneously (T2).

	Body Weight (kg)			ADG (g/Day)		
Treatment	Day 0	Day 21	Day 60	0–21 Days	21–60 Days	0–60 Days
T1—Different days	1.85	7.14	18.93	293.9	311.4	305.1
T2—Simultaneously	1.84	7.27	19.98	301.4	334	323.9
SEM	0.077	0.244	0.608	13.51	14.69	10.86
*p*-value	0.9731	0.4520	0.1061	0.3121	0.1495	0.1051

ADG: average daily gain; SEM: standard error of the mean. *p*-value: the treatment as fixed effect.

## Data Availability

Data are available upon reasonable request.
